# Mutation of the *ALBOSTRIANS* Ohnologous Gene *HvCMF3* Impairs Chloroplast Development and Thylakoid Architecture in Barley

**DOI:** 10.3389/fpls.2021.732608

**Published:** 2021-10-01

**Authors:** Mingjiu Li, Goetz Hensel, Michael Melzer, Astrid Junker, Henning Tschiersch, Hannes Ruwe, Daniel Arend, Jochen Kumlehn, Thomas Börner, Nils Stein

**Affiliations:** ^1^Genomics of Genetic Resources, Department of Genebank, Leibniz Institute of Plant Genetics and Crop Plant Research, Seeland, Germany; ^2^Plant Reproductive Biology, Department of Physiology and Cell Biology, Leibniz Institute of Plant Genetics and Crop Plant Research, Seeland, Germany; ^3^Structural Cell Biology, Department of Physiology and Cell Biology, Leibniz Institute of Plant Genetics and Crop Plant Research, Seeland, Germany; ^4^Acclimation Dynamics and Phenotyping, Department of Molecular Genetics, Leibniz Institute of Plant Genetics and Crop Plant Research, Seeland, Germany; ^5^Heterosis Research Group, Department of Molecular Genetics, Leibniz Institute of Plant Genetics and Crop Plant Research, Seeland, Germany; ^6^Molecular Genetics, Institute of Biology, Humboldt University, Berlin, Germany; ^7^Research Group Bioinformatics and Information Technology, Department of Breeding Research, Leibniz Institute of Plant Genetics and Crop Plant Research, Seeland, Germany; ^8^Department of Crop Sciences, Center for Integrated Breeding Research, Georg-August-University, Göttingen, Germany

**Keywords:** *albostrians*, chloroplast biogenesis, chloroplast translation, *Hordeum vulgare*, photosynthesis, plant phenotyping, rRNA processing, reverse genetics

## Abstract

Gene pairs resulting from whole genome duplication (WGD), so-called ohnologous genes, are retained if at least one member of the pair undergoes neo- or sub-functionalization. Phylogenetic analyses of the ohnologous genes *ALBOSTRIANS* (*HvAST/HvCMF7*) and *ALBOSTRIANS-LIKE* (*HvASL*/*HvCMF3*) of barley (*Hordeum vulgare*) revealed them as members of a subfamily of genes coding for CCT motif (CONSTANS, CONSTANS-LIKE and TIMING OF CAB1) proteins characterized by a single CCT domain and a putative N-terminal chloroplast transit peptide. Recently, we showed that *HvCMF7* is needed for chloroplast ribosome biogenesis. Here we demonstrate that mutations in *HvCMF3* lead to seedlings delayed in development. They exhibit a yellowish/light green – *xantha –* phenotype and successively develop pale green leaves. Compared to wild type, plastids of mutant seedlings show a decreased PSII efficiency, impaired processing and reduced amounts of ribosomal RNAs; they contain less thylakoids and grana with a higher number of more loosely stacked thylakoid membranes. Site-directed mutagenesis of *HvCMF3* identified a previously unknown functional domain, which is highly conserved within this subfamily of CCT domain containing proteins. HvCMF3:GFP fusion constructs were localized to plastids and nucleus. *Hvcmf3Hvcmf7* double mutants exhibited a *xantha*-albino or albino phenotype depending on the strength of molecular lesion of the *HvCMF7* allele. The chloroplast ribosome deficiency is discussed as the primary observed defect of the *Hvcmf3* mutants. Based on our observations, the genes *HvCMF3* and *HvCMF7* have similar but not identical functions in chloroplast development of barley supporting our hypothesis of neo-/sub-functionalization between both ohnologous genes.

## Introduction

Chloroplasts are the photosynthetic active type of plastids. Functional chloroplasts contain thylakoid membranes, which are the site of light-dependent photosynthesis reactions as mediated by four protein complexes – photosystem I (PSI), photosystem II (PSII), cytochrome b_6_f and ATPase (Dekker and Boekema, 2005). Thylakoid membranes appear in stacks of thylakoid disks, termed grana, and as stroma lamellae, sheets of lipid-bilayers interconnecting the grana. While PSII is mainly found in the grana thylakoids, PSI and the ATPase complex are enriched in the stroma lamellae, and the cytochrome b_6_f complex is distributed evenly between the two structures (Dekker and Boekema, 2005).

Chloroplasts originated from photosynthetic cyanobacteria (Gould et al., 2008). They contain their own genome with a core set of approximately 100 genes inherited from the cyanobacterial ancestor and possess their own machinery for gene expression, i.e., for transcription, transcript processing and translation (Börner et al., 2014; Pogson et al., 2015). Extensive studies have demonstrated that chloroplast development and function require the import of nucleus-encoded proteins; actually, more than 95% of the chloroplast proteins are encoded by the nuclear genome and subsequently targeted to the chloroplasts, in most cases with help of an N-terminal chloroplast transit peptide, cTP (Leister, 2003; Lee and Hwang, 2018).

The extant land plants exhibit very high species diversity, which is the outcome of a long-lasting evolutionary process, during which polyploidization is considered as having provided one of the major driving forces (De Bodt et al., 2005; Soltis et al., 2009; Lafon-Placette et al., 2016; Van de Peer et al., 2017; Vamosi et al., 2018). Whole genome duplication (WGD) is widespread across land plants as revealed by genome sequencing of an increasing number of species (Mühlhausen and Kollmar, 2013). After WGD genomes tend to return – through a process called diploidization – to the more stable and less redundant diploid stage. Thus, one copy of all the duplicated genes will be lost in a more or less random fashion. There are three possibilities for the evolutionary fate of duplicated genes (Lynch and Conery, 2000). In most of the cases, the function of one copy is lost either by complete deletion of the gene or through accumulating nonsense or deleterious mutations. In maize, a recent auto-polyploid, nearly half of the duplicated genes were lost during evolution (Lai et al., 2004). If both ohnologous genes are retained, one copy typically acquires a novel, beneficial function (neo-functionalization), conserved during natural selection (Lynch and Conery, 2000). The second scenario to maintain duplicated gene pairs is by sub-functionalization; each gene of an ohnologous pair partially retains the original function, but only together providing the complete functional capacity of the ancestral gene (Force et al., 1999).

A common ancestor of the family of the *Poaceae*, comprising all extant cereal crops, underwent WGD at around 70 million years ago (Paterson et al., 2004). Traces of this WGD are conserved in the barley (*Hordeum vulgare*) genome (Thiel et al., 2009) and were detected, e.g., as pairs of ohnologs among genes coding for the CCT motif family (CMF) of proteins in the genomes of cereal crops (Cockram et al., 2012). The CCT domain [from the three Arabidopsis (*Arabidopsis thaliana*) proteins CONSTANS, CONSTANS-LIKE and TIMING OF CAB1] comprises 43 amino acids and is found near the C-terminus of numerous proteins. As far as a function could be assigned, CCT domain proteins are transcription (co-) factors typically involved in modulating flowering time, light-induced signaling, circadian rhythms, or regulate the transcription of sugar inducible genes (Cockram et al., 2012).

The genes *HvCMF7* (*ALBOSTRIANS, HvAST*) and *HvCMF3* (*ALBOSTRIANS-LIKE, HvASL*) represent a pair of ohnologs within the genes coding for CMF proteins of barley (Cockram et al., 2012; Li et al., 2019). A mutation in *HvCMF7* confers the variegated “*albostrians”* phenotype (Li et al., 2019). Besides incomplete penetrance of its variegation phenotype (Hagemann and Scholz, 1962), the most prominent characteristic of the *albostrians* mutant are the ribosome-free plastids leading to albino leaves and albino sectors of striped leaves (Hess et al., 1993). The mutant served as a model to study the cross-talk between nucleus and the other DNA-containing organelles and greatly extended the field of chloroplast biology (e.g., Bradbeer et al., 1979; Hess et al., 1993; Zhelyazkova et al., 2012). The lack of plastid ribosomes and the albino phenotype of the *albostrians* mutant indicate that the presence of the wild type allele of the ohnologous gene *HvCMF3* cannot rescue the effects of the mutation in *HvCMF7* suggesting that both ohnologs do not act at complete redundancy. Strikingly, the ALBOSTRIANS protein HvCMF7 was localized to chloroplasts and potentially to the nucleus, and the phenotype of the *albostrians* mutant implies that HvCMF7 plays a role in the biogenesis and/or stability of chloroplast ribosomes, i.e., has a function and location entirely different from all previously investigated CCT domain proteins (Li et al., 2019) with the possible exception of the Arabidopsis homolog of HvCMF7 and HvCMF3, the CMF protein AtCIA2. AtCIA2 was recently reported to be also located in chloroplasts and in the nucleus (Gawroński et al., 2021). While the nuclear localization of AtCIA2 agrees with other studies, the import of AtCIA2 into plastids may need further confirmation (Sun et al., 2001; Yang and Sun, 2020; Li et al., 2021). In contrast, AtCIL (CIA2-like), the ohnolog of AtCIA2 (Mühlhausen and Kollmar, 2013) and additional Arabidopsis homolog of HvCMF7 and HvCMF3, was only detected in the nucleus (Gawroński et al., 2021; Li et al., 2021). *AtCIA2* encodes a nuclear transcription factor regulating genes for the transport of nuclear encoded proteins into chloroplasts and for the biogenesis of chloroplast ribosomes (Sun et al., 2009), a function more similar to previously published roles of investigated CCT domain proteins. Intriguingly, the *Atcia2* mutant exhibits a pale green phenotype and no indication of leaf variegation while the *Atcil* mutant resembles the wild type (Sun et al., 2001; Yang and Sun, 2020; Li et al., 2021).

Based on the ohnologous relationship between HvCMF3, ABLOSTRIANS-LIKE, and HvCMF7, ALBOSTRIANS, we aimed to test if neo-/subfunctionalization has provided the two genes with similar or distinct roles. We generated and analyzed a series of *Hvcmf3* mutants and observed a *xantha*-to-green phenotype, distinctly decreased chloroplast rRNA levels, delayed rRNA processing, altered stacking of thylakoids and reduced numbers of grana in overall smaller chloroplasts; all indicating the effect of impaired photosynthesis. HvCMF3:GFP fusions were transported to plastids and nucleus. Site-directed mutagenesis led to the identification of a highly conserved, previously unknown protein domain, which supposedly plays a key role in the determination of phenotype severity. Thus we could demonstrate that HvCMF3 and HvCMF7 are involved in related biological processes with non-redundant functions.

## Results

### Phylogenetic Relationships of *HvASL* Homologs in Monocots and Dicots

**FIGURE 1:**
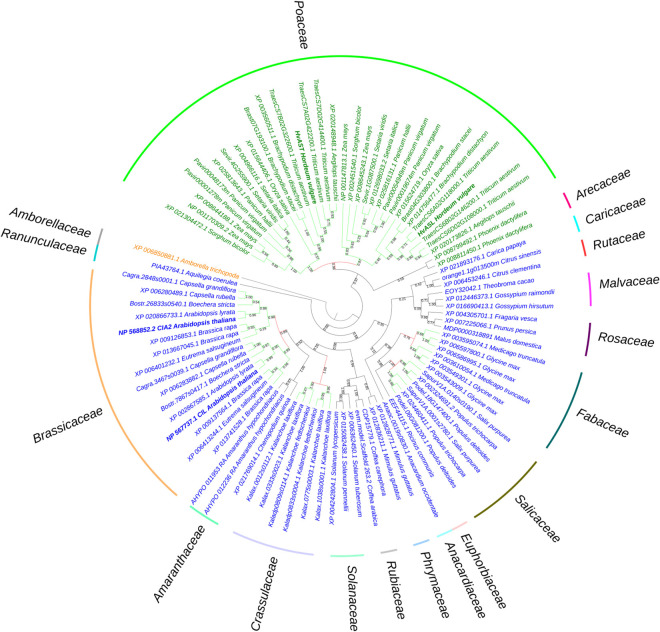
Phylogenetic analysis of *HvASL* and *HvAST* homologous genes. The phylogenetic tree shows *Amborella trichopoda* as a sister group to all other angiosperm species. The two main branches separate the monocots and dicots, indicated by green and blue color, respectively. Evolutionary analysis reveals a single pair of paralogs in diploids, and two pairs of paralogs in tetraploids. The paralogs of each species divide into two branches; each branch contains the corresponding orthologs for species in families *Poaceae*, *Salicaceae*, *Fabaceae*, *Crassulaceae*, and *Brassicaceae*. Maintenance of these paralog pairs indicates that *HvASL* probably retained an important function in barley. The numbers above/below the branches represent bootstrap values which indicate reliability of the cluster descending from that node. The red color node indicates where splitting of the orthologous groups occurred. Positions of HvASL, HvAST, AtCIL, and AtCIA2 are highlighted in bold. Family information is indicated outside the colored stripes.

The sequence of the barley genome (Mascher et al., 2017) predicts the gene model *HORVU6Hr1G021460*.*2* as the closest homolog of *HvCMF7*. We confirmed the predicted gene structure by cDNA sequencing. The gene contains three exons separated by two introns, and encodes a protein of 490 amino acids (AA) in length. Sequence comparison of *HvCMF7* and *HORVU6Hr1G021460.2* revealed that both homologs share 50.5% identity at protein level. The gene *HORVU6Hr1G021460.2* was previously designated as *HvCMF3* in a study on the evolution of the CCT domain-containing gene family (CMF) in *Poaceae* (Cockram et al., 2012). Homology searches for *HvCMF3* and *HvCMF7* against Phytozome v12.1.6 (Goodstein et al., 2012) identified a subfamily of the CMF genes comprising131 homologous genes in 66 angiosperm species, while in 14 species with an earlier evolutionary history no genes with clear homology to *HvCMF3*/*HvCMF7* could be determined. As we found a homolog also in *Amborella*, representing the most basal lineage in the clade of angiosperms (Drew et al., 2014), we used, in a further search for homologs, the *Amborella* sequence as query leading to the identification of homologous sequences also in the genomes of gymnosperms. The homologous genes were filtered by integrity and correctness of their coding sequence; as a result, 91 genes from 48 species were included in an evolutionary analysis. The maximum likelihood tree shows that *Amborella trichopoda* forms a sister clade to all the remaining angiosperm plants in accordance with previous reports (Drew et al., 2014). The monocot and dicot species separate from the main branch and form independent clades (Figure 1). Paralogous genes of all grass species in the *Poaceae* family are divided and grouped together forming two subclades. Similarly, we observed this pattern also for the dicot families *Salicaceae*, *Fabaceae*, *Crassulaceae*, and *Brassicaceae*, respectively. The conserved presence of paralogous gene pairs in grasses indicates their origin from the ancient whole-genome duplication shared among grass species (Paterson et al., 2004; Thiel et al., 2009; Cockram et al., 2012), i.e., they represent ohnologous genes (ohnologs). Interestingly, tetraploid species in the mono- and dicots, like *Panicum virgatum* and *Brassica rapa*, consistently contain two pairs of paralogs. Evidently, all ohnologs of *HvCMF3* and *HvCMF7* have been retained in the genomes of all analyzed monocot and dicot plant families, strongly suggesting that all ohnologs fulfill important functions in angiosperm plants and have non-redundant functions.

Protein alignments based on 131 HvCMF3/HvCMF7 homologs from 66 monocot and dicot species showed that the C-terminal CCT domain is conserved across all analyzed plant species. These proteins have also a putative N-terminal chloroplast transit peptide (cTP) as predicted by ChloroP (Emanuelsson et al., 1999) suggesting a role for all or most of these proteins (including the ancestor at the origin of all angiosperms) in chloroplast development and function. In the further study we aimed to make first steps in the elucidation of the biological function of the barley gene *HvCMF3 (ALBOSTRIANS-LIKE)* and to compare it with its ohnolog *HvCMF7 (ALBOSTRIANS)* and its homologs *AtCIA2* and *AtCIL*.

### *Hvcmf3* Mutant Exhibits a *xantha*-to-Green Phenotype

**FIGURE 2:**
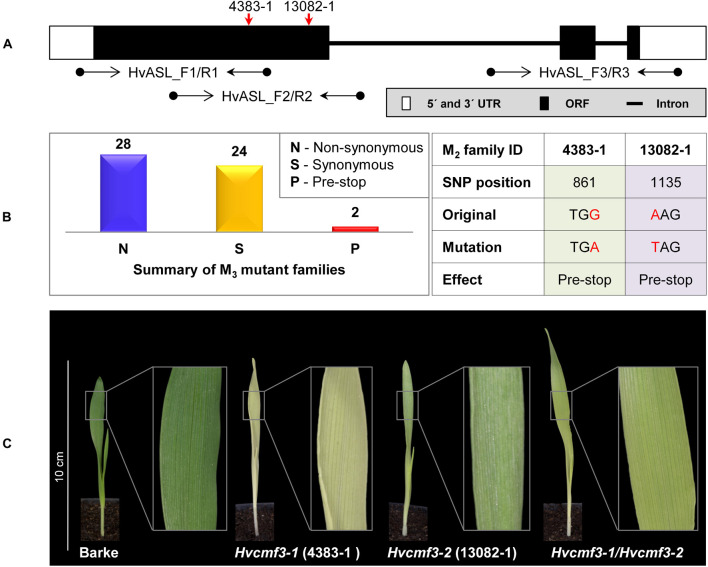
Functional validation of *HvCMF3* by TILLING and allelism test. **(A)** TILLING screening strategy. Screening of coding regions of *HvCMF3* by three primer pairs. Red arrows indicate the relative position of the stop codons of TILLING families 4383-1 and 13082-1. **(B)** Summary of the identified mutations. TILLING screening revealed a total of 54 M_3_ mutant families with lesions in the *HvCMF3* gene, including 28 non-synonymous, 24 synonymous, and 2 pre-stop mutations. Transition mutation (G to A) at position 861 results in an immature stop codon in family 4383-1. Pre-stop family 13082-1 carries a transversion mutation (A to T) at position 1135. The adenine of the *HvCMF3* start codon refers as position 1. **(C)** Phenotype of *Hvcmf3* mutants compared with wild type cv. ‘Barke’ at developmental stage 3 days after germination. Leaves of *Hvcmf3-1* mutant exhibit a *xantha* phenotype. Compared to *Hvcmf3-1*, the chlorophyll-deficient phenotype of *Hvcmf3-2* mutant is less severe. The F_1_ hybrid, *Hvcmf3-1/Hvcmf3-2* derived from crossing 4383-1 × 13082-1, exhibits a pale green phenotype.

First, we screened for mutants of *HvCMF3* by TILLING of an EMS-induced mutant population consisting of more than 7,500 M_2_ plants (Gottwald et al., 2009). Fifty-four M_2_ mutant families were identified representing 28 non-synonymous, 24 synonymous and 2 pre-stop mutations (Figures 2A,B and Supplementary Tables 1, 2) and all mutant families were assigned to phenotypic and genotypic analyses. Owing to the ohnologous relationship of *HvCMF3* and *HvCMF7*, we screened for leaf color variation in all *HvCMF3* TILLING families. We could not observe any chlorophyll-deficient phenotype in mutant families representing induced non-synonymous or synonymous single nucleotide polymorphisms. In contrast, all homozygous mutants identified at M_3_ stage of the pre-stop TILLING family 4383-1 (carries a guanine to adenine transition at nucleotide position + 861 leading to a premature stop codon) exhibited a chlorophyll-deficient *xantha*-like phenotype; while the segregating wild type and heterozygous plants of this family produced green seedlings (Figure 2C, Supplementary Figure 1, and Supplementary Table 3). The linkage was confirmed by analysis of 245 M_4_ individuals derived from nine heterozygous M_3_ plants. The phenotype of the homozygous *Hvcmf3* mutant in TILLING family 4383-1 resembles previously identified *xantha* mutants of barley (Henningsen et al., 1993), but differs from those mutants by greening of the leaves along with plant growth (Supplementary Figure 1); hence, we refer here to a *xantha*-to-green phenotype. Homozygous mutants of the second pre-stop TILLING family 13082-1 (carries a transversion from adenine to thymine at nucleotide position + 1135 leading to a premature stop codon) were identified only after propagating to the M_5_ generation. M_5_ homozygous mutants of family 13082-1 exhibit also a *xantha*-to-green phenotype. But in comparison to the pre-stop line 4383-1, they require a shorter time-span for recovery to fully green leaves (Figure 2C). The two TILLING mutant alleles of 4383-1 and 13082-1 were designated as *Hvcmf3-1* and *Hvcmf3-2*, respectively. F_1_ hybrids formed between both mutants (*Hvcmf3-1*/*Hvcmf3-2*) displayed consistently a *xantha-*to-green phenotype, thus demonstrating the allelic state of both mutations (Figure 2C), which was further confirmed by analyzing an additional 50 F_2_ plants (*Hvcmf3-1*/*Hvcmf3-2*) derived from the four F_1_ hybrids.

Based on these results we concluded that *HvCMF3*, similar to *HvCMF7*, plays a fundamental role in chloroplast development.

### Functional Validation of *HvCMF3* by Site-Directed Mutagenesis Using Cas9 Endonuclease

**FIGURE 3:**
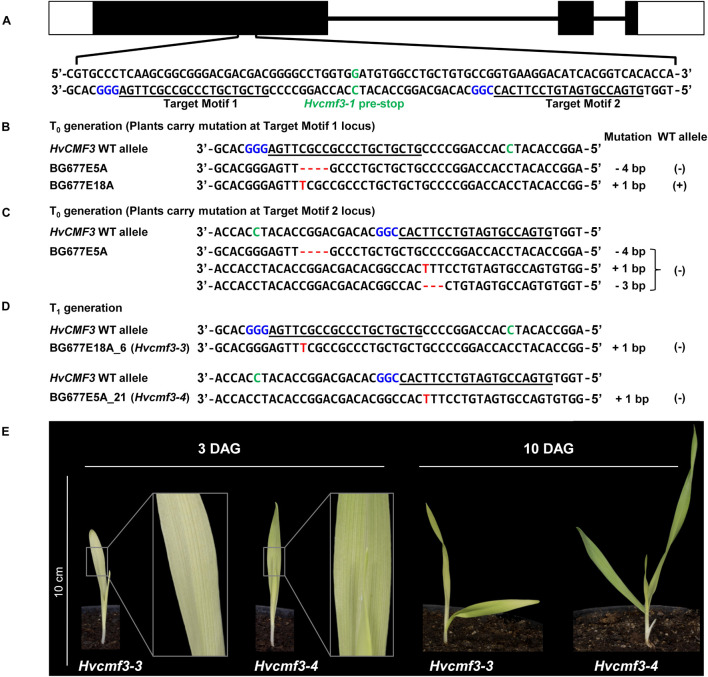
Site-directed mutagenesis of *HvCMF3* gene by RNA-guided Cas9 endonuclease. **(A)** Selection of Cas9/gRNA target sites. The two target motifs (Target Motif 1 and 2) in the anti-sense strand are underlined; the respective protospacer adjacent motif is highlighted in blue. The nucleotide in green color indicates the position of the pre-stop mutation in the *Hvcmf3-1* mutant. **(B)** Alignment of *HvCMF3* sequences of wild type and T_0_ plantlets carrying mutations at target motif 1. **(C)** Alignment of *HvCMF3* sequences of wild type and T_0_ plantlets carrying mutations at target motif 2. The chimeric and/or heterozygous T_0_ regenerant BG677E5A carries multiple mutations with each mutation shown in one single row. **(D)** Alignment of *HvCMF3* sequences of wild type and T_1_ homozygous mutant plants. Across panels, deletions are represented by red hyphens and insertions by red letters. The specific mutation of each plant is shown on the right of each sequence; presence/absence of wild type allele is indicated by symbols ±, respectively. **(E)** Phenotype of Cas9-induced homozygous *Hvcmf3* mutants at developmental stages 3 and 10 days after germination.

Remarkably, the recovery rate of *xantha*-to-green phenotype (i.e., the speed of recovery from *xantha* to green) of the *Hvcmf3-1* mutant was much slower than that of the *Hvcmf3-2* mutant. To test whether this was an effect of the different positions in the coding region of the gene of the two mutations (Supplementary Figure 3C), we adopted RNA-guided Cas9 endonuclease mediated site-directed mutagenesis in order to reproduce the position effect of phenotype severity. Two guide RNAs (gRNAs) were designed surrounding the position of the nonsense mutation of TILLING mutant 4383-1 (Figure 3A). In total, 36 primary regenerants were derived from *Agrobacterium*-mediated co-transformation of both gRNAs. Thirty-four of the 36 T_0_ plantlets carried integral T-DNA, i.e., they were PCR positive for the presence of *cas9* and the gRNA-driving *OsU3* promoter in combination with at least one gRNA (Supplementary Table 4). Among them, four plants carried both gRNAs, providing the potential of generating insertion/deletion (INDEL) mutations at the target region (Supplementary Tables 1, 4). Analysis of T_0_ plants (Figures 3B,C) (Supplementary Table 5) revealed short INDELs as the most frequent result of site-directed mutagenesis, however, larger deletions were also detected (e.g., BG677E1A, BG677E1B, and BG677E9B) (Supplementary Figure 2 and Supplementary Table 5). Sequencing of cloned PCR products revealed the chimeric state for most of the T_0_ plants. BG677E1B, however, represents a homozygous mutant. It carries a 316 bp deletion in the collected leaf sample, which showed a phenotype resembling the pre-stop TILLING mutants. Additionally, individual leaves from three independent chimeric T_0_ mutants BG677E1E, 2B and 2D, with *xantha* phenotype were confirmed to harbor frame-shift mutations and to lack the wild type allele (Supplementary Figure 2 and Supplementary Table 5). We screened eight T_1_ plants each from all of the 14 T_0_ mutant families (Supplementary Table 5) to follow transmission of the mutations through the germline. As expected, all homozygous and homogeneously biallelic mutant plants with frameshift mutations exhibited the *xantha*-to-green phenotype (Figures 3D,E and Supplementary Figure 2). It is worth noting that mutants with a lesion at target motif 1 showed a more severe phenotype than with lesions further downstream. This is not only manifested by the *xantha* leaf color variation at early developmental stage (3 DAG), but also by a slower leaf development at later stages (e.g., 10 DAG, Figure 3E). We named the mutant alleles BG677E18A_6 and BG677E5A_21, *Hvcmf3-3* and *Hvcmf3-4*, respectively. The site-directed mutagenesis experiment consolidated our previous findings by TILLING. Hence, mutations in *HvCMF3* are causal for the *xantha*-to-green mutant phenotype. Furthermore, the observed position effect of the induced mutations implies that *HvCMF3* possesses (a) further essential functional region(s) in addition to the C-terminal CCT domain, which is expected to be removed or disrupted in the proteins of all respective induced mutants.

### Identification of a Conserved Sequence Essential for HvCMF3 Function

**FIGURE 4:**
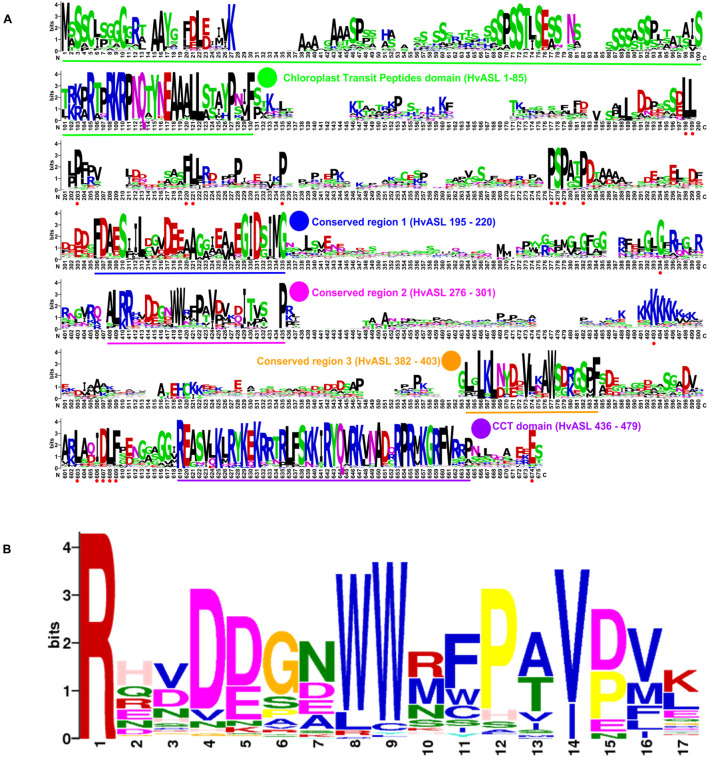
Novel conserved functional region of HvCMF3. **(A)** Alignment of 131 HvCMF3 homologous protein sequences from 66 species revealed five conserved regions which include the N-terminal chloroplast transit peptides domain, the C-terminal CCT domain and three novel conserved regions. In addition, the homologous genes contain multiple conserved peptides indicated by red dots below the position IDs. The conserved regions are marked with underline and highlighted with colored circles. The region given in parentheses indicates the corresponding position of the conserved region in reference to HvCMF3. Alignment was manually edited by removing wrongly predicted sequence regions and by filling gaps. There were a total of 675 positions left. The online tool Weblogo was adopted for graphic generation. **(B)** Conservation analysis of the functional region of HvCMF3 identified in this study. For each position, the overall height of the stack indicates the sequence conservation at that position, while the height of symbols within the stack indicates the relative frequency of each amino acid at that position.

Protein alignments of 131 HvCMF3/HvCMF7 homologs from 66 angiosperm species revealed the CCT domain near the C-terminus and, interestingly, a putative N-terminal cTP. Moreover, the proteins contain three highly conserved regions as well as further highly conserved AA residues embedded in somewhat less conserved regions without predicted function (Figure 4A). The *Hvcmf3-3* and *Hvcmf3-4* alleles differ at protein level by a truncation of 17 AA, leading to a more severe phenotype in case of *Hvcmf3-3* (Figure 3E and Supplementary Figure 3C). The missing peptide represents a conserved region, which should play an essential functional role in the protein (conserved region 2 in Figure 4A). In an attempt to test this hypothesis, we screened T_1_ regenerants carrying both gRNAs with the expectation to observe large deletions extending over the identified conserved region. We identified four homozygous plants with in-frame deletion from mutant family BG677E9B. All exhibit the *xantha*-to-green mutant phenotype; among them, one with 57 bp and another three with 51 bp deletions. Since none of the deletions affected the splicing site they are expected to result in 19 and 17 AA deletions, respectively, at protein level (Supplementary Figures 3, 4). The mutant allele with a 51 bp deletion is designated as *Hvcmf3-5*. Two homozygous mutants (new allele *Hvcmf3-6*), carrying a 19 bp deletion combined with a 34 bp insertion, were identified in family BG677E2C (Supplementary Figure 3). This mutation led to the substitution of seven AA at position 290–296 (PAVPVKD) by 12 AA (HSTDATARTGSG) (Supplementary Figure 3D). The *Hvcmf3-6* mutant showed a green (wild type) phenotype indicating that replacement of the seven original AA (PAVPVKD) did not affect HvCMF3 protein function. We performed conservation analysis for the deleted region in *Hvcmf3-5* by comparing 116 homologous sequences from 59 angiosperm species as described in section “Materials and Methods.” The first AA ‘R’ (i.e., arginine) is 100% conserved among all 116 sequences (Figure 4B). As revealed by the substitution mutant *Hvcmf3-6* in family BG677E2C, the C-terminal six AA (Figure 4B, positions 12–17) have no effect on HvCMF3 protein function. Therefore, the peptide of AA 279–289 (Figure 4B, positions 1–11) represents a previously unknown conserved functional region within the conserved domain 2. Neither the identified novel functional region nor the entire conserved domain 2 of HvCMF3 is reported in the NCBI’s Conserved Domain Database (Marchler-Bauer et al., 2017).

### Reduced Chloroplast Ribosome Accumulation in *Hvcmf3* Mutants

**FIGURE 5:**
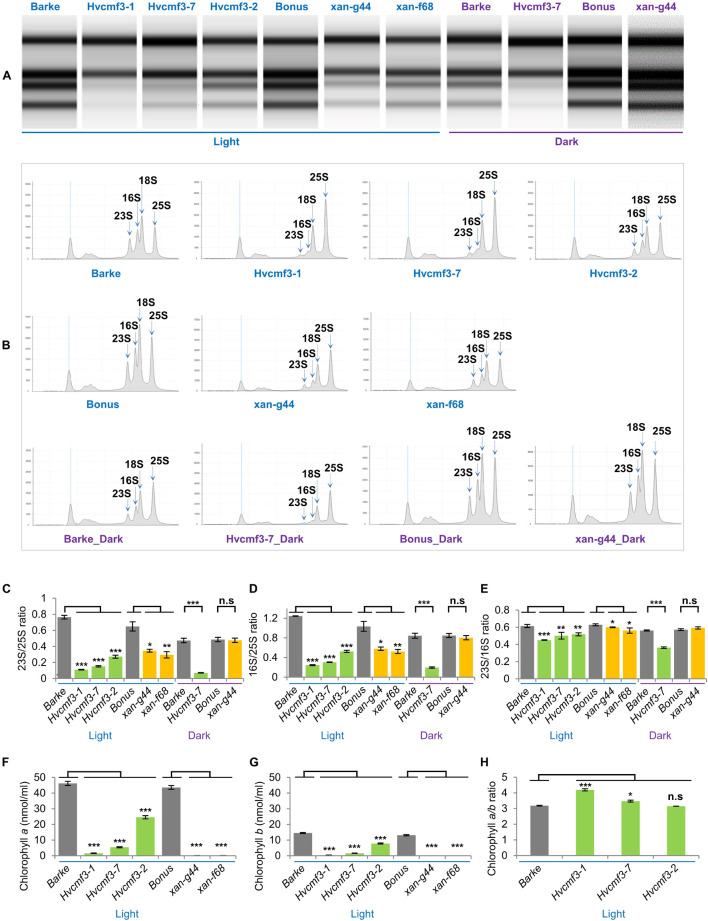
rRNA analysis and chlorophyll content measurement. **(A)** Separation of cytosolic and plastid rRNAs using the Agilent high sensitivity RNA ScreenTape assay. **(B)** Analysis of rRNA from wild type, *Hvcmf3* mutants and *xantha* mutants using an Agilent Tapestation 4200. **(C,D)** Determination of plastid-to-cytosolic rRNA ratios. **(C)** 23S/25S; **(D)** 16S/25S. **(E)** Ratio of the plastid 23S rRNA to the plastid 16S rRNA. **(F–H)** Analysis of chlorophyll contents and ratio between chlorophyll *a* and chlorophyll *b*. Results are presented as means ± SE. *t-test* significant level: *p < 0.05; **p < 0.01; ***p < 0.001, n.s, not significant. Three plants per genotype were analyzed. The level of significance is given for paired comparisons of either genotype within the two categories. The exact *p*-values are provided in Supplementary Dataset 3).

One of the most prominent characteristics of the *albostrians* mutant is the lack of ribosomes in plastids of albino leaves and albino sections of striped leaves (Hess et al., 1993; Li et al., 2019). We checked therefore whether mutation of *HvCMF3* has also an effect on plastid ribosomes. The accumulation of rRNA levels can be used as a proxy for ribosomal subunit accumulation (Walter et al., 2010). Thus, we quantified chloroplast and cytosolic rRNA fractions in light- and dark-grown seedlings of *Hvcmf3* mutants. Due to the *xantha*-to-green phenotype of young *Hvcmf3*, we compared *Hvcmf3* with the previously described barley *xantha* mutants, *xan-g44* and *xan-f68*, which contain only trace amounts of chlorophyll in their leaves due to defects in the magnesium chelatase (EC 6.6.1.1) subunits D and H, respectively (Olsson et al., 2004; Axelsson et al., 2006). This enzyme catalyzes the insertion of magnesium into protoporphyrin IX, the first unique step of the chlorophyll biosynthetic pathway (Figures 5A,B). The relative abundance of chloroplast to cytosolic ribosomal subunits was determined by their ratios. Under light condition, *Hvcmf3* mutants as well as *xan-g44* and *xan-f68* have reduced amounts of both large (50S) and small subunits (30S) of the plastid ribosomes, as indicated by the lower 23S:25S and 16S:25S ratios, respectively (Figures 5B,C). It should be noted that the 23S rRNA contains a so-called hidden break and is therefore represented by two smaller RNAs in monocots, one of them shows a migration behavior similar to the 18S rRNA and is thus not visible as a separate band and one below the 16S rRNA (Figure 5B). The lower level of plastid rRNAs in light-grown *xan-g44* and *xan-f68* is a secondary effect of the low chlorophyll content and accumulation of chlorophyll precursors. Under these conditions, light leads to the production of ROS (Reactive Oxygen Species) in the plastids and consequently to the degradation of plastid rRNAs and low levels of plastid ribosomes (e.g., Willi et al., 2018). Interestingly, while dark-grown *xan-g44* and *xan-f68* exhibit wild type levels of plastid rRNAs, the dark-grown *Hvcmf3* mutant [*Hvcmf3-7* (Supplementary Figure 5D), exhibiting an ivory phenotype] has very low plastid rRNA levels indicating that the low content of plastid rRNA in the *Hvcmf3* mutant is not caused by light-induced degradation but is a direct effect of the mutation (Figures 5B–D). Consistent with the reduced amount of plastid rRNA, the chlorophyll content in the *Hvcmf3* mutants is significantly decreased compared to the wild type (Figures 5F,G). Mutant *Hvcmf3-1*, which exhibits the most severe phenotype, shows a higher chlorophyll *a:b* ratio than wild type barley (Figure 5H). As PSII is enriched in chlorophyll *b* as compared to PSI, the higher chlorophyll *a:b* ratio may indicate that PSII is more severely affected than PSI in mutant *Hvcmf3-1* (Figure 5H). Nevertheless, the higher chlorophyll *a:b* ratio ameliorates during the greening process as evidenced by mutants *Hvcmf3-7* and *Hvcmf3-2*, suggesting that deficits in biogenesis of the photosynthetic complex can be compensated over time.

### HvCMF3 Is Required for Maturation of 16S and 23S rRNAs

**FIGURE 6:**
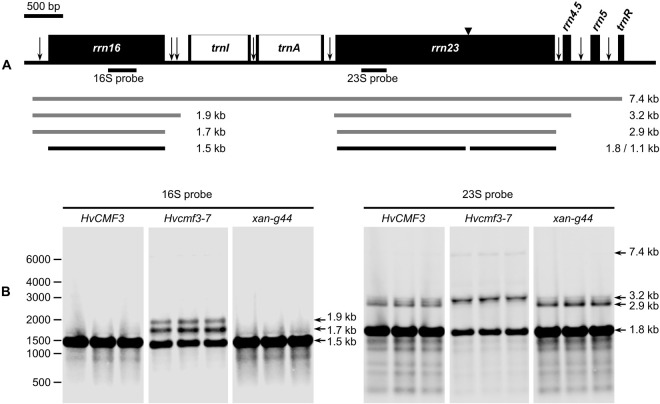
Analysis of 16S and 23S rRNA processing in wild type, *Hvcmf3-7* mutant and *xan-g44* mutant. **(A)** Schematic representation of the chloroplast *rrn* operon in barley. Black boxes indicate exons and white boxes indicate introns. Vertical arrows indicate processing sites in the primary transcripts of the *rrn* operon. Positions of an internal cleavage site (hidden break) in the 23S rRNA is shown as black triangle. Positions of the hybridization probes for 16S and 23S are indicated below the operon structure. The 7.4-kb primary transcript and various processing precursors are shown with gray lines; the mature forms of 16S and 23S rRNAs are shown with black lines. **(B)** Analysis of 16S and 23S rRNA processing by RNA gel-blot. Numbers on the leftmost indicate positions of the marker bands. Arrows point to various forms of mature and precursor rRNAs mentioned in the text and shown in **(A)**.

Chloroplast rRNA genes are cotranscribed with tRNAs in the order 16S-*trnI-trnA*-23S-4.5S-5S-*trnR* (Figure 6A), and efficient processing of this precursor is essential for maintaining chloroplast translation. As shown above, accumulation of plastid 16S and 23S rRNAs is reduced in *Hvcmf3* mutants (Figure 5). Several mutants with impaired chloroplast translation, e.g., due to ribosome deficiency, show defects in the processing of plastid RNAs (e.g., Barkan, 1993; Yu et al., 2008; Jiang et al., 2018). To determine if reduced rRNA levels are correlated with defects in rRNA processing in *Hvcmf3-7*, RNA gel-blot analysis was performed on RNAs isolated from the 5 cm basal parts of primary leaves of 10-day-old wild type (*HvCMF3*), *Hvcmf3-7* and *xan-g44* with specific probes against the 16S and 23S rRNAs, respectively (Figure 6B). The existence of mature rRNAs demonstrates that, in principle, all processing steps starting from the primary transcript are functioning. However, both 16S and 23S rRNAs exhibit inefficient processing in the *Hvcmf3-7* mutant; while the processing patterns did not differ between *xan-g44* and wild type. Thus, it can be ruled out that the inefficient processing in the *Hvcmf3-7* mutant was caused by secondary effect due to chlorophyll deficiency and impaired photosynthetic activity. The 1.9 and 1.7 kb precursors were detected in the *Hvcmf3-7* mutant. The more abundant 1.7 kb compared to 1.9 kb RNA suggests that the last processing step leading from the 1.7 kb precursor to the mature 1.5 kb form is particularly slow in the *Hvcmf3-7* mutant, whereas all processing steps between the primary transcript and the 1.9 kb precursor seem to be comparably fast in *Hvcmf3-7* since these precursor transcripts are not detected in the mutant like in the wild type. A very weak primary transcript is, however, detected by the 23S probe in *Hvcmf3-7*, but not in wild type and *xan-g44* suggesting a somewhat slower activity also of the first steps of plastid rRNA processing (Figure 6B). The *Hvcmf3-7* mutant also showed impaired processing of 23S species by overaccumulation of the 3.2 kb precursor and less abundant mature 23S species (Figure 6B), indicating inefficient separation of the 23S and 4.5S RNAs (Figure 6). In contrast, the absence of the intact 2.9 kb 23S rRNA and the detection of the final 1.8 kb processing products (Figure 6B) suggest that also the *Hvcmf3-7* mutant efficiently cuts the RNA at position of the “hidden break” (Kössel et al., 1985). Overall, maturation of 16S and 23S rRNAs is inefficient in the absence of HvCMF3.

### Mutation of *HvCMF3* Affects Photosynthesis

**FIGURE 7:**
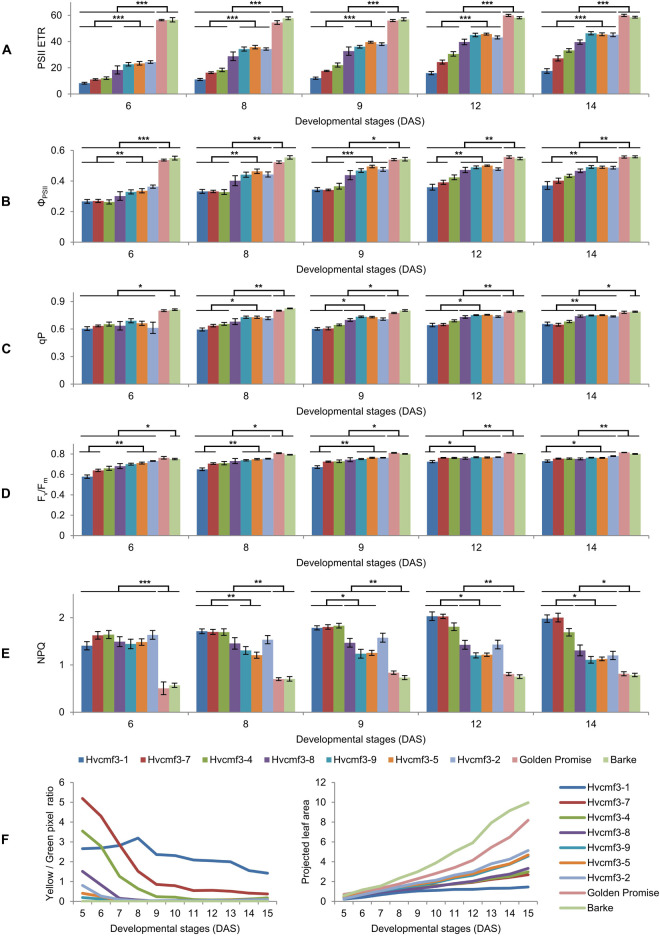
Determination of photosynthetic parameters and growth dynamics of *Hvcmf3* mutant and wild type control plants. **(A–E)** Measurement of photosynthetic parameters during early developmental stages. Results are presented as means ± SE. *Student’s t-test* significant levels, ^∗^*p* < 0.05; ^∗∗^*p* < 0.01, ^∗∗∗^*p* < 0.001. Four to eight plants per genotype were analyzed. The level of significance is given for paired comparisons of either genotype within the two categories. The raw data of the LemnaTec traits is provided in Supplementary Dataset 4, and *p*-values are provided in Supplementary Dataset 5. ETR, electron transport rate; Φ_PSII_, photosystem II operating efficiency; qP, fraction of PSII centers that are ‘open’ based on the puddle model; F_v_/F_m_, maximum quantum yield of PSII photochemistry measured in the dark-adapted state; NPQ, non-photochemical quenching. **(F)** Plant growth dynamics. Left panel is yellow/green pixel ratio, and right panel is projected leaf area.

Because of the plastid ribosome deficiency (Figure 5), the *Hvcmf3* plants potentially suffer from insufficient levels of protein synthesis in chloroplasts. Since part of the proteins of all components of the photosynthetic apparatus are being synthesized on plastid ribosomes, the efficiency of photosynthetic electron transport can serve as a highly sensitive indicator of plastid translational capacity (Rogalski et al., 2008). PSII is known to require a particularly high translation capacity due to the constant requirement for repair synthesis of the D1 protein (Takahashi and Badger, 2011). To test this, we quantified photosynthesis-related traits in a series of *Hvcmf3* mutants with different severity of their pigment-deficiency phenotype by using a chlorophyll fluorescence imaging-based method integrated into an automated, conveyor-based phenotyping platform (Junker et al., 2015). Initially, 96 plants from 12 families, each with 8 replicates, were sown (Supplementary Figure 5 and Supplementary Table 6). After filtering the non- or badly-germinated seeds and the chimeric seedlings, 60 plants were left for analysis including seven mutant and two wild type families, respectively, each with four to eight replicates (Supplementary Table 6). Based on the severity of phenotype, the nine plant families were classified into three groups: Group I: wild type (Barke and Golden Promise); Group II: mutant families 4383-1 (*Hvcmf3-1*), BG677E2A_2 (*Hvcmf3-7*) and BG677E5A_21 (*Hvcmf3-4*); and Group III: BG677E5A_19 (*Hvcmf3-8*), BG677E9B_1 (*Hvcmf3-9*), BG677E9B_6 (*Hvcmf3-5*) and 13082-1 (*Hvcmf3-2*) (Supplementary Figure 5D). Consistent with the reduced amount of plastid rRNAs, the PSII electron transport rate (ETR) is lower in the mutants compared to wild type. Moreover, the ETR of Group II mutants is significantly lower than of Group III (Figure 7A). The quantification of PSII operating efficiency (ΦPSII) of light-adapted plants revealed a lower PSII yield of the mutants compared to the wild type during early developmental stages (i.e., 6–14 DAS). Moreover, PSII operating efficiency of the two mutant groups also showed significant difference to each other (Figure 7B). Another parameter, qP, which represents the proportion of PSII reaction centers that are open, was significantly lower in the *Hvcmf3* seedlings than in the wild type (Figure 7C). In line with the decreased ΦPSII, the maximum quantum efficiency of PSII (F_v_/F_m_) was also significantly reduced in *Hvcmf3* (Figure 7D). In contrast to the lower PSII yield, a higher proportion of excitation energy was released in *Hvcmf3* as thermal dissipation compared to the wild type (Figure 7E). Group II mutants showed higher levels of non-photochemical quenching (NPQ) compared to Group III mutants (Figure 7E). The distinct PSII ETR and PSII operating efficiency levels were also reflected by the different severity of the phenotype (Figure 7F). In line with the reduced chlorophyll contents of the *Hvcmf3* mutants (Figures 5F–H), quantification of the plant coloration revealed that Group II mutants have higher yellow/green pixel ratio compared to Group III mutants. Due to their slower development, Group II mutants exhibited smaller overall projected leaf areas than Group III mutants and wild type (Figure 7F). Taken together, this data demonstrates that *Hvcmf3* mutants show a lower PSII activity which correlates with the reduced levels of plastid rRNA, i.e., mutants with the lowest plastid rRNA levels showed also the lowest PSII efficiency and the lowest PSII ETR. This data supports our hypothesis of *Hvcmf3* mutants suffering from impaired chloroplast translation and that the observed impact on PSII is most likely a consequence of the plastid ribosome deficiency and not a direct effect of the mutations.

### Mutation of *HvCMF3* Affects Chloroplast Development and Grana Organization

**FIGURE 8:**
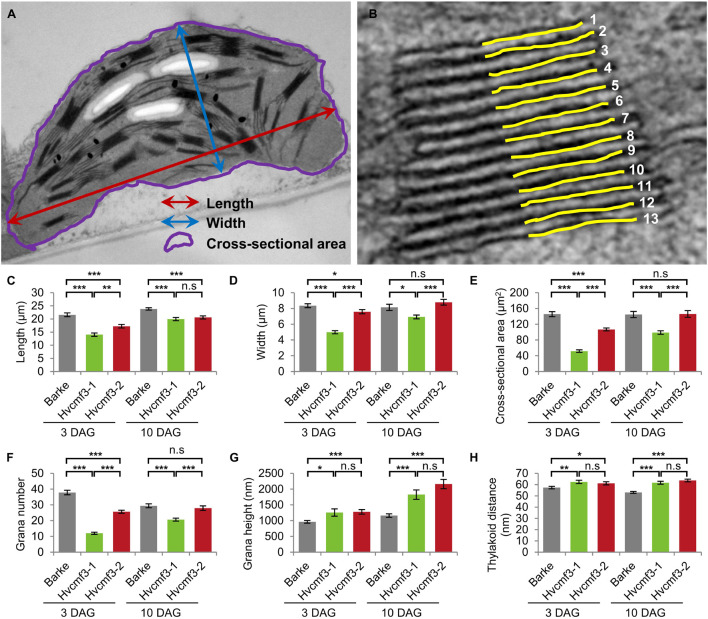
Quantification of chloroplast architecture components. **(A)** Diagram for demonstrating the chloroplast length, width, and surface area. **(B)** Illustration demonstrating the counting of thylakoid. **(C–H)** Comparison of chloroplast morphology and grana architecture between wild type and *Hvcmf3* mutants at developmental stages 3 days after germination. Chloroplast length **(C)**, chloroplast width **(D)**, chloroplast cross-sectional area **(E)**, grana number **(F)**, grana height **(G)**, and thylakoid distance **(H)**. Results are presented as means ± SE. *t-test* significant level: *p < 0.05; **p < 0.01; ***p < 0.001, n.s, not significant. Number of chloroplast analyzed *n* ≥ 24. The exact *p*-values are provided in Supplementary Dataset 6.

To clarify if the *xantha*-to-green phenotype of *Hvcmf3* is only manifested in physiological or also in anatomical changes, we analyzed leaf samples of the pre-stop TILLING mutants *Hvcmf3-1* and *Hvcmf3-2* at two developmental stages (3 and 10 days after germination, DAG) by transmission electron microscopy (TEM) (Supplementary Figures 6, 7). Mesophyll cells of mutant *Hvcmf3-1* contained smaller chloroplasts than both the wild type and mutant *Hvcmf3-2* at 3 DAG and 10 DAG (Supplementary Figure 7). At 3 DAG, the chloroplast size of *Hvcmf3-2* was also reduced in comparison to wild type (Supplementary Figures 7A–F). At 10 DAG, the chloroplast size in *Hvcmf3-2* was indistinguishable from wild type, while *Hvcmf3-1* still contained smaller chloroplasts (Supplementary Figures 7G–L). Compared with wild type chloroplasts, both mutants showed a distinct difference in the structure of their grana, which (at least partially) were build up by a higher number of thylakoids with less condensed stacking at both developmental stages (Supplementary Figure 7). Next, we performed quantitative assessments for chloroplast length, width and surface area, as well as grana number, the extent of grana stacking and distance between thylakoid membranes within the grana (Figures 8A,B). In both mutants, chloroplasts are smaller than in wild type leaves at 3 DAG as determined by the parameter ‘surface area’ (Figures 8C–E). Chloroplast size was also significantly different (Student’s *t*-test, *p* = 6.4 × 10^–15^) between *Hvcmf3-1* and *Hvcmf3-2*, which correlates well with the difference in phenotype severity between *Hvcmf3-1* and *Hvcmf3-2* at 3 DAG (Figures 2C, 8C–E). At 10 DAG, the development of chloroplast shape and morphology of mutant *Hvcmf3-1* remained delayed. In contrast, although chloroplast length of mutant *Hvcmf3-2* was still reduced if compared to the wild type, chloroplast width and surface area approached the wild type level (Figures 8C–E). *Hvcmf3* mutations influence also grana organization. At 3 DAG, chloroplasts of both TILLING mutants contained lower numbers of grana stacks (Figure 8F). In contrast to *Hvcmf3-2*, the number of grana was significantly reduced (Student’s *t*-test, *p* = 6.8 × 10^–15^) in chloroplasts of *Hvcmf3-1*, also at 10 DAG (Figure 8F). The observed increased grana stacking in both mutants is a result of a higher number of thylakoids and of enhanced distances between thylakoid membranes within the stacks (Figures 8G,H and Supplementary Figure 8). In summary, the analyzed *Hvcmf3* mutants are affected in PSII efficiency and ETR, which is underpinned by severe anatomical changes: smaller than wild type chloroplasts containing a lower number of thylakoids and larger but loosely stacked grana.

### HvCMF3 Is Targeted to Plastids and Nuclei

**FIGURE 9:**
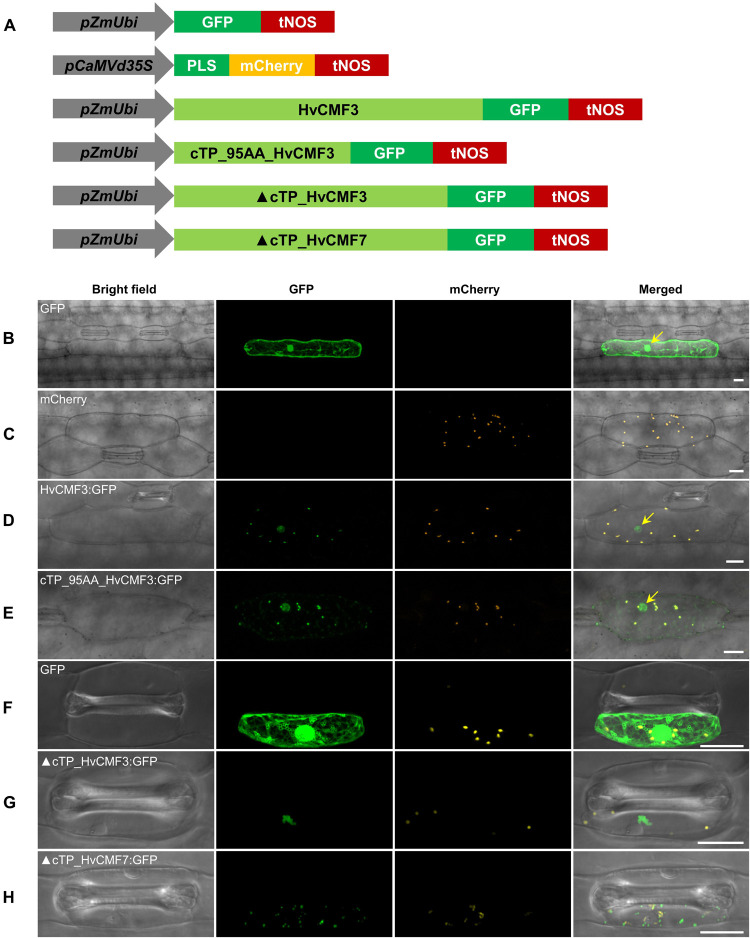
Subcellular localization of HvCMF3. **(A)** Schematic diagram of the constructs prepared for transient expression. *pZmUbi*, maize *UBIQUITIN1* promoter. *pCaMVd35S*, Cauliflower Mosaic Virus doubled-enhanced *35S* promoter. GFP, green fluorescent protein. mCherry, mCherry fluorescent protein; PLS, plastid localization signal, i.e., the chloroplast transit peptide (N-terminal 79 amino acids) of the small subunit of tobacco RUBISCO. HvCMF3, coding sequence of wild type *HvCMF3* gene. cTP_95AA_HvCMF3, N-terminal chloroplast transit peptide of HvCMF3 with a length of 95 amino acids as predicted by online tool PredSL. ▲cTP_HvCMF3, HvCMF3 without N-terminal cTP (T2-T95). ▲cTP_HvCMF7, HvCMF7 without N-terminal cTP (A2-A83). tNOS, *Agrobacterium nopaline synthase* terminator. The schematic drawing is not in proportion with gene length. The first leaf of 7-day-old barley seedlings was used for particle bombardment. The fluorescence was checked 24 h after bombardment. Scale bar for all images is 20 μm. **(B)** Localization of GFP control with *GFP* being driven by the maize UBIQUITIN1 promoter. **(C)** Localization of the plastid marker. **(D)** Localization of HvCMF3:GFP. The GFP fluorescence signal is targeted both to plastid and nucleus. **(E)** Localization of cTP_95AA_HvCMF3:GFP. The yellow arrows in the merged panels indicate the nucleus. **(F–H)** Localization of GFP control **(F)**, ▲cTP_HvCMF3 **(G)**, and ▲cTP_HvCMF7 **(H)**. Both truncated HvCMF3 and HvCMF7 form aggregates in the cytoplasm.

Similar to its ohnolog HvCMF7 (Li et al., 2019), *in silico* analysis by PredSL (Petsalaki et al., 2006) predicted the presence of a 95 AA chloroplast transit peptide at the N-terminus of HvCMF3 (also other *in silico* tools predict chloroplast location, Supplementary Table 7). To test its function, we studied the transient subcellular localization of green fluorescent protein (GFP) fusion constructs with either the complete wild type *HvCMF3* allele (HvCMF3:GFP) or the putative cTP of HvCMF3 only (cTP_95AA_HvCMF3:GFP) (Figure 9A and Supplementary Table 7). GFP fused to wild type HvCMF3 accumulated in the plastids of epidermis cells, co-localizing with mCherry, the chloroplast location control (Figure 9D). A plastid location was also observed for the cTP_95AA_HvCMF3:GFP construct confirming the functionality of the predicted cTP at the N-terminal domain of HvCMF3 (Figure 9E). *In silico* tools predict also a nuclear localization of HvCMF3 (Supplementary Table 7). As previously observed with HvCMF7 (Li et al., 2019), GFP fluorescence of the HvCMF3:GFP construct was indeed additionally found in the nucleus (Figure 9D). Also free GFP is in the nucleus. There are many reports in the literature describing an identical distribution of GFP in the cytoplasm and nucleus. The likely reason for the observed nuclear location is that GFP is small enough to allow diffusion not only through the cytoplasm but also into the nucleus without a specific transport mechanism (von Arnim et al., 1998). In contrast, the HvCMF3:GFP fusion protein is too large to enter the nucleus by diffusion and expected to contain one or more NLS. That the observed nuclear location of HvCMF3 is not an artifact is further supported by differences in the distribution of free GFP and the HvCMF3:GFP construct: free GPF is observed all over the cytoplasm and evenly distributed over the nucleus. In contrast, the HvCMF3:GFP fusion protein (Figure 9D) and also GFP fused to the predicted cTP of HvCMF3 (Figure 9E) do not accumulate in the cytoplasm but specifically in the plastids and the nucleus. Moreover, the fusion product with HvCMF3 is not evenly distributed but shows regions of higher and lower fluorescence within the nuclei (Figures 9D,E). We made the same observations with HvCMF7 (Li et al., 2019). The observed specific distribution of GFP to plastids and nuclei when fused to the predicted cTPs of HvCMF3 and HvCMF7 suggests the presence of NLS in these N-terminal regions of the proteins. To obtain further support for a nuclear localization of HvCMF3, we used the cNLS software (Kosugi et al., 2009) to screen for nuclear localization signals (NLS) in HvCMF3. Interestingly, the *in silico* analysis detected region R58-P91 as NLS (cutoff score = 6.2) which resides within the cTP region. The software predicts also NLS in this highly conserved region for HvCMF7, AtCIA2 and AtCIL (Li et al., 2021). Interestingly, both truncated HvCMF3:GFP (▲cTP_HvCMF3:GFP; i.e., HvCMF3 without N-terminal cTP: T2-T95) and truncated HvCMF7:GFP (▲cTP_HvCMF7:GFP; i.e., HvCMF7 without N-terminal cTP: A2-A83) cannot enter chloroplasts and nuclei, but form aggregates in the cytoplasm (Figures 9F–H). This observation provides additional support for the proposed dual targeting of HvCMF3 and HvCMF7 to plastids and nucleus with help of their N-terminal sequences.

### *Hvcmf3/Hvcmf7* Double Mutant Exhibits a Mixed *xantha*-Albino Variegation Phenotype

**FIGURE 10:**
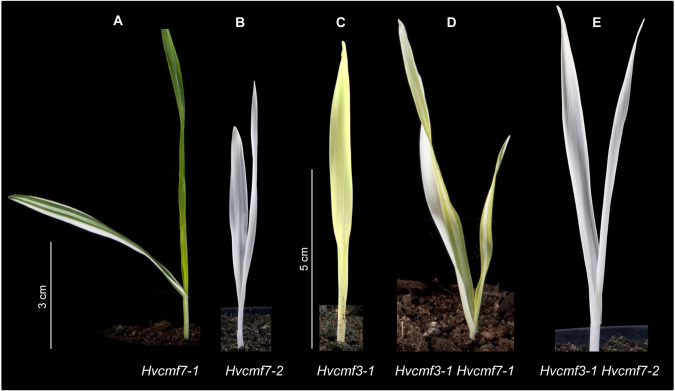
Phenotype of double mutant *Hvcmf3/Hvcmf7*. **(A)** The original *albostrians* mutant *Hvcmf7-1* shows a green-white striped phenotype. **(B)** The *albostrians* pre-stop TILLING mutant *Hvcmf7-2* exhibits a complete albino phenotype. **(C)** Phenotype of *Hvcmf3-1* mutant at 7 days after germination. **(D)** Double mutant *Hvcmf3-1 Hvcmf7-1* shows a *xantha*-albino striped phenotype. **(E)** Double mutant *Hvcmf3-1 Hvcmf7-2* exhibits albino phenotype. Scale bars: **(A,B)** 3 cm; **(C–E)** 5 cm.

Our results revealed that mutation of either of the ohnologs *HvCMF3* and *HvCMF7* is causing a chlorophyll-deficient phenotype. While *Hvcmf3* mutants exhibit a *xantha*-to-green recovery phenotype, *Hvcmf7* mutants show either a green-white variegation or a complete albino phenotype (Li et al., 2019). Both genes are essential for normal chloroplast development. Mutation of *HvCMF3* reduces the amount of plastid ribosomes, affects chloroplast size and the morphology of grana stacks while *HvCMF7* mutants do not show any development of chloroplasts and possess only proplastid-like ribosome-free plastids in their mesophyll cells. Homozygous *Hvcmf3-1/Hvcmf7-1* double mutants derived from crossing *Hvcmf7-1* × *Hvcmf3-1* showed a *xantha*-albino striped phenotype (Figure 10). If the more severe *Hvcmf7-2* mutant 6460-1 was used as a crossing parent (Li et al., 2019), the resulting homozygous double mutant *Hvcmf3-1/Hvcmf7-2* exhibited always the complete albino phenotype of *Hvcmf7-2* (Figure 10). We interpret the observed morphological, functional and cell biological differences of *Hvcmf3* and *Hvcmf7* mutants as a manifestation of the neo-subfunctionalization of both genes since their occurrence through WGD.

## Discussion

Plastid-encoded proteins are mainly involved in plastid gene transcription and translation or are playing a role in photosynthesis. Most of the genes needed for plastid functions and in particular for the development of chloroplasts and their photosynthetic apparatus are, however, encoded in the nuclear genome and are targeted to the plastid/chloroplast; including genes involved in chloroplast transcription, RNA processing, RNA stability, and translation (Börner et al., 2014; Pogson et al., 2015). Of specific interest are nuclear encoded genes which are dually targeted to both plastids and nucleus since they might be involved in the regulation of chloroplast biogenesis and in the communication between nucleus and plastids (Krupinska et al., 2020). Here we report on first studies toward the function of *HvCMF3.* With this initial characterization of *HvCMF3* mutants, it is possible to compare potential functions of four proteins belonging to the family of genes coding for CCT motif (CMF) proteins (Cockram et al., 2012): ALBOSTRIANS (HvAST/HvCMF7), ALBOSTRIANS-LIKE (HvASL/HvCMF3), CHLOROPLAST IMPORT APPARATUS 2 (AtCIA2/AtCMF14) and CHLOROPLAST IMPORT APPARATUS 2-LIKE (AtCIL/AtCMF9). All four proteins share a very similar structure with a putative N-terminal cTP, several conserved domains of unknown function (the functional importance of one conserved region has been demonstrated in the present study), additional conserved amino acids and the CCT domain near the C terminus. The highly conserved sequence and domain organization strongly suggest similar functions for these proteins. Indeed, mutation of their genes leads consistently to impaired chloroplast development and affects chloroplast ribosomes as reported earlier (Sun et al., 2001, 2009; Li et al., 2019) and in this report. In case of *cil*, the mutational effect becomes obvious only in the state of double mutant *cia2cil* (Gawroński et al., 2021; Li et al., 2021).

### HvCMF3 Belongs to a Small Subfamily of CCT Domain Proteins

Numerous CCT-containing genes represent transcription factors that regulate gene expression in the nucleus through DNA-binding or by integration into DNA-binding protein complexes (Wenkel et al., 2006; Jang et al., 2008). Based on their domain structure, CCT proteins may be classified into COL (CONSTANS-LIKE) proteins having one or two zinc-finger B-Box domains, PRR (PSEUDO RESPONSE REGULATOR) proteins with a pseudo response regulator domain, and CMF (CCT MOTIF FAMILY) proteins containing only the CCT domain and lacking other known functional domains (Cockram et al., 2012). Both, HvCMF3 and HvCMF7, carry only a CCT domain and no other characterized functional domain. Thus, they are assigned to the CMF family, which comprises nine genes in barley (Cockram et al., 2012). In the present study as well as in our previous work on the characterization of HvCMF7 (Li et al., 2019), we demonstrate that HvCMF3 and HvCMF7 share an N-terminal cTP and the C-terminal CCT domain, but, in contrast to other CMF domain proteins, carry additional, previously uncharacterized conserved regions; one of them proved to be essential for wild type gene function in the present study. This domain structure including a putative cTP is shared by the Arabidopsis homologs of HvCMF3 and HvCMF7, AtCMF14 (AtCIA2), and AtCMF9 (AtCIL), and by homologous proteins in the other families of angiosperms and gymnosperms (Figure 1). Based on the three more intensively studied genes/proteins of this CMF gene sub-family, we propose to differentiate them from other CMF genes by assigning them to a new CMF sub-family, the AAC proteins [for: ALBOSTRIANS/HvCMF7 (Li et al., 2019), ALBOSTRIANS-LIKE/HvCMF3, CHLOROPLAST IMPORT APPARATUS 2/AtCIA2 (Sun et al., 2009)]. According to the phylogenetic tree of CCT domains (Cockram et al., 2012), these genes form a branch in a subclade of clade 2. Clade 2 comprises CMF genes/proteins characterized by a specific position of an intron within the gene region coding for the CCT domain (Cockram et al., 2012).

### HvCMF3 Potentially Plays a Role in Chloroplast Ribosome Formation/Maintenance

We observed a very low amount of chloroplast rRNA in leaves with low chlorophyll content in *Hvcmf3* mutants at early developmental stages. Both chlorophyll and chloroplast rRNA content improved with further development, however, without reaching wild type level. A further striking feature of *HvCMF3* mutants are the dramatic changes in the internal structures of chloroplasts with a decreased number of thylakoids and at the same time larger and more loosely stacked grana. Although we cannot rule out other functions of HvCMF3, we regard the observed chloroplast rRNA deficiency as the most likely primary observed effect of the studied *Hvcmf3* mutants and all other observed effects of the mutations as being caused by the chloroplast translation deficiency. One reason for this conclusion is that similar phenotypes have previously been described for many mutants with reduced chloroplast translation. Although the phenotypes are different in details and highly variable depending on the type of mutated gene, on the severity of the translation deficiency, and on the phase of chloroplast development, when the translation deficiency starts to become effective, all mutants with impaired chloroplast translation show pigment deficiencies, lower performance of photosynthesis and altered thylakoid organization, often combined with retarded growth and delayed greening (Albrecht et al., 2006; Delannoy et al., 2009; Tiller and Bock, 2014; Liu et al., 2015; Kohler et al., 2016; Aryamanesh et al., 2017; Zhang et al., 2017).

Another reason for proposing the ribosome deficiency as the primary effect among the observed effects is that pigment deficiency, altered thylakoid organization or impaired photosynthesis does not cause chloroplast ribosome deficiencies, while the opposite occurs and can be explained by the function of chloroplast translation (Fristedt et al., 2014; Zoschke et al., 2017). Chloroplast genes encode essential components of the photosynthetic apparatus including subunits of PSI, PSII, Cytb_6_f, ATP synthase and NDH, i.e., these proteins are synthesized on chloroplast ribosomes. Thus, a reduced amount of chloroplast ribosomes, as observed in *HvCMF3* mutants, will negatively affect photosynthesis and will also have effects on thylakoid architecture. In this context it is interesting to note that the formation of large grana was observed in a barley mutant lacking PSII reaction centers (Simpson et al., 1989) and in Arabidopsis plants treated with the chloroplast translation inhibitor lincomycin (Belgio et al., 2015).

Chloroplast ribosome deficiency as the reason of the phenotype of *Hvcmf3* plants is also supported by the fact that the severity of ribosome deficiency is correlated with increasingly drastic effects on chlorophyll content, PSII efficiency, and grana morphology. Plastid ribosome deficiency can have various reasons. Mutation of nuclear genes, e.g., for ribosomal proteins, translation factors, RNA processing factors and others will impair the biogenesis and function of plastid ribosomes. It remains to be investigated whether the observed inefficient RNA processing of *Hvcmf3* may lead to the plastid ribosome deficiency or whether, *vice versa*, ribosome-deficiency and/or limited capacity of translation may impair RNA processing. Our observation of a delayed plastid rRNA processing in *Hvcmf3*, while *xantha* mutants with a similar phenotype and reduced plastid rRNA content did not show indications of defective RNA processing, suggest a more specific role of HvCMF3 in plastid rRNA processing.

HvCMF3 and HvCMF7 might have similar functions. The functions of HvCMF3 and HvCMF7 are, however, not identical as can be expected when two ohnologs have been retained in the genome for a period of about 70 million years since the WGD they originate from. We deduce non-identical functions for *HvCMF3* and *HvCMF7* from our observation that the genes cannot replace each other in mutants. Moreover, the mutants of *HvCMF3* and *HvCMF7* have clearly different phenotypes. While mutation of *HvCMF7* results in an albino phenotype, the lack of ribosomes and, consequently, in a complete stop of chloroplast development, *Hvcmf3* mutants show a *xantha*-to-green phenotype, possess plastid ribosomes, although distinctly reduced in their number, and show a retarded chloroplast development. Interesting phenotypic differences were revealed in double mutants depending on the involved *Hvcmf7* allele. All seedlings of the *Hvcmf7-*2 mutant show an albino phenotype and lack plastid ribosomes (Li et al., 2019). Crossing of *Hvcmf7-2* with *Hvcmf3-1* resulted in double mutants exhibiting the *Hvcmf7-2* albino phenotype. This, however, is not surprising, if the function of both proteins is needed to reach the normal number of ribosomes and the malfunction of one alone (HvCMF7) is already sufficient to cause the complete loss of ribosomes and the complete stop of chloroplast development, that is, more effect is not possible. A different result was obtained when crossing the original *albostrians* mutant (*Hvcmf7-1*) with *Hvcmf3-1*. Homozygous plants carrying the *Hvcmf7-1* allele are characterized by green-white striped leaves. Although the green sectors of variegated leaves are, like the white sectors, homozygous for the mutant *Hvcmf7-1* allele, their phenotype is indistinguishable from the wild type. They contain normal chloroplasts with no indication for a ribosome deficiency. A threshold effect was discussed to explain the variegation (Li et al., 2019). The double mutant *Hvcmf3-1/Hvcmf7-1* has, like *albostrians Hvcmf7-*1, variegated leaves with white stripes. However, instead of the green stripes observed in *albostrians*, the double mutant has yellow-green stripes, i.e., exhibit the *xantha* phenotype typical for *Hvcmf3-1*. This result suggests that there is no interaction between HvCMF3-1 and HvCMF7-1, which would inhibit the expression of the *Hvcmf3-1* phenotype. Further studies are needed to analyze the expression of the two genes and their mutant versions during leaf and chloroplast development.

### HvCMF3 and Its Homologs as Nuclear Proteins

The localization of HvCMF3 and HVCMF7 in plastids would fit to their proposed role in the formation and/or maintenance of plastid ribosomes, but the exact function of these proteins in plastids remains to be further investigated.

The situation is more complex since our data strongly suggest a dual localization of HvCMF3 and HvCMF7 in plastids and nuclei (Figure 9; Li et al., 2019). Thus, in addition to their function in plastids/chloroplasts, they are expected to play a – possibly different – role in the nucleus. The Arabidopsis homolog AtCIA2 was recently reported to be dually targeted to plastids and nucleus (Gawroński et al., 2021) while AtCIL has been found only in the nucleus (Yang and Sun, 2020; Gawroński et al., 2021; Li et al., 2021). All four AAC proteins share the same domain structure. The function of the cTP domain as mediator of the transport of the protein into plastids has been confirmed for HvCMF3 (this study), HvCMF7 (Li et al., 2019) and AtCIA2 (Gawroński et al., 2021). However, the conserved N-terminal sequence is larger than expected for a role only as cTP (Figure 4). Lack of the predicted cTPs impairs not only the transit of HvCMF3 and HvCMF7 into plastids but prevents also their transport into the nucleus (Figure 9). This is in agreement with the *in silico* predicted presence of NLS in the N-terminal sequence of these proteins. Yang and Sun (2020) identified experimentally an NLS in the N-terminal sequence of AtCIA2 and AtCIL. This sequence is also conserved in HvCMF3 and HvCMF7 and is likely the NLS that supports the observed import of the barley proteins into nuclei (Figure 9). While this N-terminal NLS was sufficient for transport of AtCIL into nuclei, AtCIA2 needed a second NLS, a K-rich sequence more in the middle of the protein (Yang and Sun, 2020). As we found GFP not only in the plastids but also in the nuclei (but not in the cytoplasm) when fused to the predicted transit peptides of HvCMF3 or HVCMF7, their N-terminal NLS likely are also sufficient for import into the nuclei as in case of AtCIL.

The CCT domain has been studied in nuclear transcription factors and reported to act as NLS, to serve DNA binding as well as protein-protein interactions (Kurup et al., 2000; Strayer et al., 2000; Robson et al., 2001; Shen et al., 2020; Yang and Sun, 2020). Yang and Sun (2020) found no evidence for a role of the CCT domain as NLS for AtCIA2 and AtCIL, and also our observations suggest that not the CCT domain but an N-terminal sequence functions as the NLS for HvCMF3 and HvCMF7 (Figure 9). The CCT domain is lacking or disrupted in the mutants analyzed in this study. Nevertheless, all *Hvcmf3* mutants, even those that are expected to have altered or lacking amino acid sequences starting closer to the N-terminus, i.e., more distant from the CCT domain than in the original *albostrians* mutant (*Hvcmf7-1)*, show a mild phenotype compared to *albostrians* and resemble more the *Atcia2* and *Atcil* mutants. The drastic effect of mutations of *HvCMF7* on the phenotype is caused most likely by the lack of plastid ribosomes, a defect which fully blocks chloroplast development and the formation of the photosynthetic apparatus. Nevertheless, also *Hvcmf3, Atcia2*, and *cia2cil* mutants show reduced amounts of plastid rRNA and impaired plastid rRNA processing (this study; Gawroński et al., 2021; Li et al., 2021). The earlier observation of reduced transcript accumulation of nuclear genes coding for ribosomal proteins and components of the chloroplast protein import apparatus in *Atcia2* (Sun et al., 2009), which has recently been confirmed for genes encoding plastid ribosomal RNAs by RNASeq analysis (Gawroński et al., 2021), may be an effect of transcriptional regulation by AtCIA2 (and AtCIL) binding together in complex with other regulators at the promoter region of the respective genes (Sun et al., 2009). Also, other genes involved in chloroplast development might be regulated in this way and their impaired transcription might contribute in addition to the impaired translation to the phenotype of mutations in AAC genes. Notably, Gawroński et al. (2021) reported altered response of *cia2* and *cia2cil* to certain abiotic stresses.

However, chloroplast ribosome/translation deficiencies have not only effects on chloroplast development via the reduced synthesis of chloroplast-gene encoded proteins, but also via plastid-to-nucleus retrograde signaling. Plastid-to-nucleus signaling affects the expression of numerous nuclear genes coding for proteins with roles in photosynthesis, thylakoid formation, and pigment synthesis, but also nuclear genes involved in the response to biotic and abiotic stresses as first observed in the *albostrians* mutant (Hess et al., 1994, 1998; for review see Kleine and Leister, 2016; Crawford et al., 2018; Dietz et al., 2019; Wu and Bock, 2021). Thus, one should keep in mind that part of the observed effects of mutations of AAC genes on the expression of nuclear genes for chloroplast biogenesis, photosynthesis and stress response could be indirect effects due to retrograde signaling. The dual targeting of ACC proteins into nuclei and plastids is particularly interesting and could be related to a hypothetical role in retrograde signaling (Krupinska et al., 2020). Mutant analyses including the present report have provided evidence for an essential role of AAC proteins in chloroplast development and more specifically in the function and biogenesis of chloroplast ribosome biogenesis. Obviously, there is need for more investigations into other suggested functions of these proteins in particular in chloroplasts but also in the nucleus. More studies on directed point mutations in the conserved regions and the search for interacting molecules also within plastids will further elucidate the functions of these proteins.

## Materials and Methods

### Plant Material and Growth Conditions

M_3_ TILLING families carrying single nucleotide polymorphisms (SNP) causing non-synonymous or pre-stop mutations were selected for phenotyping. For each family 16 plants were characterized phenotypically and further genotyped for the respective *HvCMF3* alleles via either Sanger sequencing or CAPS assay. The barley cultivar ‘Golden Promise’ was used for generation of the transgenic lines. The primary T_0_ plantlets were grown in a climate chamber with long day condition (16 h light/8 h dark; constant temperature 22°C) until reaching the third-leaf stage and then transferred to a greenhouse with the same photoperiod regime but variable day/night temperature 20°C/15°C. Supplemental light (300 μmol photons m^–2^ s^–1^) was used to extend the natural light with incandescent lamps (SON-T Agro 400; MASSIVE-GROW, Bochum, Germany). All TILLING mutants and *xantha* mutants were grown under the same greenhouse condition as the transgenic lines. For dark treatment, grains were germinated within a carton box wrapped with aluminum foil under the greenhouse condition.

For automated phenotyping, after 24 h imbibition on water-soaked filter paper, germinated grains were transferred to 10 cm pot (diameter) filled with a mixture of 85% (v) red substrate 1 (Klasmann-Deilmann GmbH, Geeste, Germany) and 15% (v) sand. All the plants were grown under controlled conditions at 20/16°C under a circadian rhythm 16-h light/8-h darkness, 70% relative humidity, photosynthetic active radiation (PAR) of 300 μmol photons m^–2^ s^–1^ in the growth chamber. In total, 96 plants including 12 genotypes each with 8 replicates were phenotypically evaluated under the LemnaTec Scanalyzer system (LemnaTec AG, Aachen, Germany) at the IPK Gatersleben. The 12 genotypes consist of two TILLING mutant lines 4383-1 (*Hvcmf3-1*; M5 lines) and 13082-1 (*Hvcmf3-2*; M6 lines); eight *Cas9*-induced T2 mutant lines BG677E1B_3, BG677E2A_2 (*Hvcmf3-7*), BG677E5A_2, BG677E5A_21 (*Hvcmf3-4*), BG677E5A_19 (*Hvcmf3-8*), BG677E9B_1 (*Hvcmf3-9*), BG677E9B_6 (*Hvcmf3-5*), and BG677E18A_6 (Supplementary Figure 5), and the two wild type cultivars ‘Barke’ and ‘Golden Promise,’ which represent the genetic background of the TILLING and *Cas9*-induced mutants, respectively.

### Phylogenetic Analysis

The barley ALBOSTRIANS protein sequence was used as BLASTP query to retrieve homologs from other species on NCBI and phytozome (Goodstein et al., 2012) databases. Phylogenetic analysis was performed using MEGA6 (Tamura et al., 2013) following the protocol of Hall (Hall, 2013). The alignment method MUSCLE was chosen to build the alignment. During the subsequent sequence validation process, the aligned sequences were manually edited by removing wrongly predicted sequence regions and filling gaps. Wrongly predicted sequence regions refer to obviously mis-predicted sequences with large insertions that are not shared with any of the aligned sequences. Sequences derived from low-coverage genomes often contain many gaps. Automatic alignment software is not aware of these gaps and thus often generates global alignments instead of local alignments with gap regions. We maintained the integrity of exons preceding and following these gaps, and added alignment gaps accordingly. Information about the sequence alignment is given in Supplementary Dataset 1. The 91 species included in phylogenetic analysis is provided in Supplementary Dataset 2, including information on homologous sequences precluded from final analysis). Next, phylogenetic tree construction was performed based on the Maximum Likelihood (ML) statistical method. The Bootstrap method with 1,000 Bootstrap Replications was set to estimate reliability of the phylogenetic tree. The Jones-Taylor-Thomton (JTT) model and Gamma Distributed (G) were selected for options Model/Method and Rates among Sites, respectively. The gaps were treated with partial deletion option i.e., all positions containing gaps and missing data less than 95% coverage were eliminated. There were a total of 264 positions in the final dataset. The phylogenetic tree was visualized with iTOL (Letunic and Bork, 2016).

### TILLING Screening

In an effort to identify *HvCMF3* mutated alleles, an EMS-induced TILLING population (Gottwald et al., 2009) was screened by placing three primer pairs to cover the coding regions of the *HvCMF3* gene (Supplementary Tables 1, 2) and mutations were detected as described previously (Li et al., 2019). Phenotypic and genotypic analyses were performed with the M_3_ progeny of the identified M_2_ families, which carried non-synonymous or pre-stop mutations. The two pre-stop TILLING families, 4383-1 and 13082-1, were further propagated and analyzed in M_4_ and M_5_ generations to confirm the linkage between the genotype of the *HvCMF3* locus and the observed phenotype.

### Site-Directed Mutagenesis Using Cas9 Endonuclease

Targeted mutagenesis using Cas9 endonuclease was adopted to generate mutations in the *HvCMF3* gene. In the first step, the ‘KNOCKIN’ tool on Deskgen Cloud was chosen for guide RNA (gRNA) design^1^. The coding sequence of *HvCMF3* was used as query and two proper gRNA target motifs were selected surrounding the position of the pre-stop mutation of TILLING mutant 4383-1. The predicted gRNA activity scored 50 and 58 for target motif 1 (3′-GGGAGTTCGCCGCCCTGCTGCTG-5′) and target motif 2 (3′-GGCCACTTCCTGTAGTGCCAGTG-5′), respectively. Both target motifs were located at the antisense strand and the underlined nucleotides represent the protospacer adjacent motif (PAM). Next, the *HvCMF3*-specific protospacer sequences were synthesized by introducing proper overhangs to facilitate downstream cloning steps (gRNA1 forward: 5′-GGCGTCGTCGTCCCGCCGCTTGA-3′ and reverse: 5′-AAACTCAAGCGGCGGGACGACGAC-3′; gRNA2 forward: 5′-GGCGTGACCGTGATGTCCTTCAC-3′ and reverse: 5′-AAACGTGAAGGACATCACGGTCAC-3′). The protospacer sequence (i.e., annealed oligonucleotides) was then cloned into vector pSH91 (Budhagatapalli et al., 2016). The derived vector was designated as pGH379-7 for gRNA1 and pGH380-12 for gRNA2. Subsequently, the expression cassette of pGH379-7 and pGH380-12 was transferred into the binary vector p6i-d35S-TE9 (DNA-Cloning-Service, Hamburg, Germany) through *Sfi*I cloning sites. The resulting plasmids pGH449-2 and pGH450-6 were co-transformed into barley cv. ‘Golden Promise’ following a previously established protocol (Hensel et al., 2009). To check for T-DNA integration in regenerated T_0_ plantlets, PCR primers targeting the *hpt* or *cas9* gene and the *OsU3* promoter were used in PCR reactions (Supplementary Table 1). Besides, presence/absence of gRNA1 and/or gRNA2 of each plant were verified by protospacer-specific primers (Supplementary Table 1). Primer pair HvCMF3_F2/R2 was employed to detect mutations for the pre-selected target regions of *HvCMF3*. Mutations carried by the chimeric T_0_ plants were further characterized by sub-cloning PCR products using the CloneJET PCR cloning Kit (Thermo Scientific, Wilmington, DE, United States); at least eight colonies were sequenced. T_0_ plants with mutations were further propagated to T_1_ generation. In analogy to analysis of the T_0_ plants, inheritance of the mutations was checked for T_1_ progenies. Additionally, T_1_ plants were phenotyped in terms of its leaf color variation during developmental stages of the initial three leaves.

### *HvCMF3* Gene Structure Analysis

The structure of the *HvCMF3* gene was determined by analysis of its cDNA. Total RNA was extracted from leaf material of a 3-day-old barley seedling (cv. Barke) using the Trizol reagent (Thermo Scientific, Wilmington, DE, United States) following the manufacturer’s instructions. Concentration of the RNA is measured by help of a NanoDrop 1000 spectrophotometer (Thermo Scientific, Wilmington, DE, United States) and further diluted to 1 μg/μL for downstream application. The prepared RNA was first treated with RNase-free DNase I (Fermentas, St. Leon-Rot, Germany) to remove potential DNA contamination; then used for cDNA synthesis applying the SuperScript^TM^ III First-Strand Synthesis System Kit (Thermo Scientific, Wilmington, DE, United States) following the manufacturer’s instructions. Next, RT-PCR was performed using primers that cover the *HvCMF3* coding regions (Supplementary Table 1) as previously described (Li et al., 2019). RT-PCR products were purified using the NucleoFast^®^ 96 PCR Kit (Macherey-Nagel, Düren, Germany) and Sanger sequenced on an ABI 3730 XL platform (Life Technologies GmbH, Darmstadt, Germany). The *HvCMF3* exon-intron-structure was revealed by alignment of the coding sequence to the corresponding genomic region.

### Cleaved Amplified Polymorphic Sequences Assay

One Cleaved Amplified Polymorphic Sequences (CAPS) marker was developed for genotyping the two *HvCMF3* pre-stop TILLING mutants, respectively. Briefly, PCR reactions were performed as described earlier (Li et al., 2019) with minor changes, i.e., the annealing temperature for the touch-down profile was 62°C to 57°C instead of 65°C to 60°C. The SNP carrying by the PCR amplicon was converted into a CAPS marker by help of the SNP2CAPS software (Thiel et al., 2004) for the selection of the proper restriction enzyme (Supplementary Table 3). Differentiation of the genotypes was achieved by the distinct digestion patterns resolved on 1.5% (w/v) agarose gels (Invitrogen GmbH, Darmstadt, Germany).

### Identification of Conserved Sequence Regions

For conservation analysis, all identified 131 *HvCMF3*-homologous sequences were aligned using MEGA6 with the MUSCEL method (Tamura et al., 2013). Information about the sequence alignment is provided in Supplementary File 1. Conservation of the resulting 675 aligned positions was displayed by the online tool WebLogo (Crooks et al., 2004).

For conservation analysis of the novel functional region identified in this study, the conserved region 2 was extracted from the above aligned file and then re-aligned in MEGA6 with the MUSCEL method (Tamura et al., 2013). Next, sequences with unequal length compared to the prominent motif (17 AA in length) were eliminated. Finally, 116 sequences from 59 species with a consistent 17 AA length were obtained. Peptide conservation was visualized using the online tool MEME (Bailey et al., 2009).

### Ribosomal RNA Analysis

Primary leaf tissue (6 cm from the top) was collected from 10-day-old seedlings. RNA isolation and determination of RNA concentration were performed as previously described (Li et al., 2019). In short, an Agilent 4200 TapeStation System (Agilent, Santa Clara, CA, United States) was adopted for analysis of rRNA. Initially, the concentration of the RNA was determined by help of a Qubit^®^ 2.0 Fluorometer (Life Technologies GmbH, Darmstadt, Germany) according to manufacturer’s instructions. RNA samples were further diluted within a quantitative range of 1–10 ng/μL. RNA quality and quantity was then measured using an Agilent High Sensitivity RNA ScreenTape following the manufacturer’s manual (Agilent, Santa Clara, CA, United States).

### RNA Gel-Blot Analysis

Primary leaf tissue (5 cm from the bottom) was collected from 10-day-old seedlings. One microgram of total RNA was separated in a 1.2% agarose/formaldehyde gel. RNA was transferred to Hybond-N (GE Healthcare) by passive transfer overnight in 25 mM sodium phosphate buffer. Membranes were UV cross-linked and hybridized in Ambion^®^ ULTRAhyb^®^ at 65°C overnight with fluorescently labeled RNA probes generated by *in vitro* transcription using templates generated by PCR using oligonucleotides described in Supplementary Table 1. The *in vitro* transcription reaction contained 5-Azido-C3-UTP (Jena Bioscience). Purified RNA probes were Click-labeled with either Cy5.5-alkyne or Cy7.5-alkyne (Lumiprobe). Hybridized membranes were washed twice in 0.5X SSC and twice in 0.1X SSC at 65°C. Membranes were scanned using the Odyssey CLx Imaging system (LI-COR).

### Chlorophyll Content Measurement

Leaf material was collected from primary leaves of 10-day-old seedlings. Samples were weighted and then frozen in liquid nitrogen. After homogenization using Mixer Mill MM400 (Retsch GmbH, Haan, Germany), 1.5 mL of *N,N*-Dimethylformamide (DMF) was added to each sample, followed by mixing on an overhead shaker (Keison Products, Chelmsford, England) for 30 min. Subsequently, the supernatant obtained after centrifugation (14,000 × *g* for 10 min, room temperature) was transferred to a new 2 mL Eppendorf tube. Chlorophyll content measurement and calculation were performed according to Porra et al. (1989). In brief, cuvette-based measurement (cuvette with 1 mm path length) was conducted by help of the Spectramax Plus spectrophotometer (GENEO BioTechProducts GmbH, Germany). Chlorophyll content of *a* and *b* was calculated by the following equation: chlorophyll *a* = 13.43(*A*^663.8^ - *A*^750^) - 3.47(*A*^646.8^ - *A*^750^); chlorophyll *b* = 22.90(*A*^646.8^ - *A*^750^) - 5.38(*A*^663.8^ - *A*^750^).

### High-Throughput Automated, Imaging-Based Phenotyping

Phenotyping by RGB (Red Green Blue, i.e., visible light) and static fluorescence imaging as described in Junker et al. (2015) started at 5 DAS and was thereafter performed daily until 14 DAS. Kinetic chlorophyll fluorescence measurements were performed using the integrated FluorCam imaging fluorimeter (Photon Systems Instruments, Brno, Czechia). Chlorophyll fluorescence kinetics was measured following a protocol optimized for the automated high throughput imaging system (Tschiersch et al., 2017). Measurement of PSII operating efficiency (Φ_PSII_) and ETR were performed with light adapted plants. For adaptation, plants were incubated in the adaptation tunnel for 5 min followed by 1 min illumination after moving into the chlorophyll fluorescence imaging (CFI) chamber with equal light intensity of 300 μmol photons m^–2^ s^–1^. Subsequently, a saturating flash with PAR (photosynthetic active radiation) intensity 4100 μmol photons m^–2^ s^–1^ for a period of 800 ms was applied to induce maximal chlorophyll fluorescence (F_m_’). The steady state fluorescence emission (F’) and F_m_’ were recorded by the FluorCam imaging module. The formula Φ_PSII_ = (F_m_’-F’)/F_m_’ was used to calculate effective quantum yield of photochemical energy conversion in PSII. The ETR was calculated as ETR = ΦPSII × PAR × 0.5 × *ABS* where PAR equals 300 in this study, 0.5 is a factor that accounts for the fraction of excitation energy distributed to PSII, and the factor *ABS* (Absorbance) represents the leaf absorbance as determined by the near-infrared (NIR) and red light (RED) sources. It is calculated by the equation *ABS* = (NIR-RED)/(NIR + RED). The PSII operating efficiency was measured at the time points 6, 7, 8, 9, 12, and 14 DAS.

Quenching parameters were determined during the night when plants were dark-adapted in the growth chamber for at least 2 h. The minimal chlorophyll fluorescence intensity (F_0_) was measured after moving into the CFI chamber and the maximal chlorophyll fluorescence intensity (F_m_) was induced by application of a saturating flash (4100 μmol photons m^–2^ s^–1^) for 800 ms. After 10 s in darkness, plants were illuminated with actinic light (300 μmol photons m^–2^ s^–1^) for 4 min. During the quenching procedure, a saturating flash was applied for 9 s after application of the actinic light and repeated 6 times with an interval of 46 s. The values of maximal chlorophyll fluorescence intensity F_m_’ and steady state fluorescence emission F’ were collected from the last saturating flash when the plants were light-adapted. Non-photochemical quenching (NPQ) was calculated using the equation NPQ = (F_m_/F_m_’)-1; and photochemical quenching (qP) using the equation qP = (F_m_’-F’)/(F_m_’-F_0_’). The distance between the FluorCam panels and plants was set to 27 cm. The quenching experiment was performed at 6, 8, 9, 12, and 14 DAS.

From daily RGB and static fluorescence imaging, amongst others the traits ‘projected leaf area’ and yellow to green pixel ratio were extracted after automated image pre-processing and segmentation using the Integrated Analysis Platform (Klukas et al., 2014). Both parameters were measured based on images acquired from the side view. These traits are a proxy for plant growth dynamics during the phenotyping experiment and the dynamics of plant coloration and the *xantha*-to-green phenotype during early seedling development, respectively. To comply with the FAIR principles of data management, the phenotyping procedures and dataset have been described using standardized metadata formats (Rocca-Serra et al., 2010) following the recommendations of the Minimum Information About a Plant Phenotyping Experiment version 1.1 (MIAPPE v1.1) recommendations (Cwiek-Kupczynska et al., 2016) and the entire dataset comprising raw and result image data as well as derived phenotypic trait tables and metadata descriptions was uploaded to the Plant Genomics and Phenomics repository (Arend et al., 2016) using the e!DAL data publication pipeline (Arend et al., 2014).

### Chloroplast Ultrastructural Analysis

Primary leaves of two developmental stages (3 and 10 days after germination) were collected from wild type Barke, mutant 4383-1 and mutant 13082-1. For comparative ultrastructural analysis, leaf cuttings of a size of 1 × 2 mm from corresponding regions (Supplementary Figure 6) of three biological replicates were used for combined conventional and microwave assisted chemical fixation, substitution and resin embedding as defined in the given protocol (Supplementary Table 8). Sectioning and TEM analysis was performed as described (Daghma et al., 2011).

### Subcellular Localization

Two constructs, HvCMF3:GFP and cTP_95AA_HvCMF3:GFP, were used to investigate the subcellular localization of HvCMF3. For HvCMF3:GFP, the coding sequence of cv. ‘Barke’ was amplified using cDNA as a template employing the manually designed primer pair HvCMF3_SC_F/HvCMF3_SC_R with *Spe*I and *Hin*dIII restriction sites introduced at the 5′ and 3′ end, respectively. Similarly, primer pair HvCMF3_ cTP_95AA_F/HvCMF3_cTP_95AA_R with restriction sites as mentioned above was used to amplify the HvCMF3 cTP predicted by the online tool PredSL (Petsalaki et al., 2006; Supplementary Tables 1, 7). The derived PCR fragments were separately inserted into vector pSB179 (Li et al., 2019). The resulting vectors HvCMF3:GFP and cTP_95AA_HvCMF3:GFP were investigated for transient expression in barley epidermal cells via biolistic assay by using the PDS-1000/He Hepta^TM^ device (Bio-Rad, Munich, Germany). A plastid marker pt-rk CD3-999 containing the *mCherry* gene driven by the doubled enhanced *CaMV 35S* promoter was adopted for particle co-bombardment with the *HvCMF3* constructs (Plastid marker TAIR link^2^). Four to six primary leaves were harvested from 7-day-old seedlings and placed on 1% Agar supplemented with 20 μg/mL benzimidazol and 10 μg/mL chloramphenicol. Gold suspension was prepared by suspending 30 mg gold particles (diameter = 1.0 μm, Bio-Rad, Munich, Germany) in 1 mL 100% ethanol. For each shooting, 50 μL of gold suspension was taken and washed three times with 100 μL ddH_2_O followed by suspension in 25 μL ddH_2_O. Then, gold particles were coated with 5 μL of plasmids (2.5 μL each of HvCMF3 construct and plastid marker; both with a concentration of 1 μg/μL) in the presence of 25 μL 25 mM CaCl_2_ and 10 μL 0.1 M spermidine under vortexing for 2 min. After centrifugation, the plasmid-gold-pellet was washed twice with 100% ethanol and suspended in 60 μL 100% ethanol. A total of 5 μL of plasmids-coated gold suspension was loaded onto each of seven macro-carriers pre-washed with 100% ethanol and dried under a fume hood. Plasmids pSB179 and pt-rk CD3-999 were bombarded individually with 1100 psi acceleration pressure and 27 inch Hg vacuum pressure in controls for distribution pattern of GFP and mCherry fluorescence, respectively. The biolistically transformed leaves were incubated at room temperature for 24 h followed by detection of the fluorescent signals by help of a Zeiss LSM780 confocal laser scanning microscope (Carl Zeiss, Jena, Germany). Green fluorescence of GFP was visualized by using the 488 nm excitation laser line with a manually defined 490–530 bandpass; mCherry signals were detected by the 561 nm excitation laser in combination with a 580–620 nm bandpass.

### Crossing Experiments

Allelism tests between *Hvcmf3-1* and *Hvcmf3-2* were performed by crossing TILLING mutant 4383-1 (maternal parent) with TILLING mutant 13082-1 (pollen donor). F_1_ hybrids carrying both mutant alleles were phenotypically characterized during the first to three leaf stages. Generation of *Hvcmf3/Hvcmf7* double mutants was achieved by crossing TILLING mutant 4383-1 as pollen donor with heterozygous *albostrians* TILLING mutant 6460-1 and the original *albostrians* mutant M4205, respectively. F_1_ plants heterozygous for both *HvCMF3* and *HvCMF7* loci were kept and *Hvcmf3/Hvcmf7* double mutants were further selected in F_2_ generation.

## Data Availability Statement

The datasets presented in this study can be found in online repositories. The names of the repository/repositories and accession number(s) can be found below: The complete phenomics dataset (images, trait values, and metadata) has been deposited in e!DAL – The Plant Genomics and Phenomics Research Data Repository. Link to the data: http://dx.doi.org/10.5447/ipk/2021/18.

## Author Contributions

ML, NS, TB, and JK conceived the study. ML, GH, MM, AJ, HT, and HR performed the experiments. AJ and DA contributed phenotyping data. ML, AJ, HT, and HR analyzed the data. ML coordinated the project. ML, NS, and TB wrote the manuscript. All authors contributed to the article and approved the submitted version.

## Conflict of Interest

The authors declare that the research was conducted in the absence of any commercial or financial relationships that could be construed as a potential conflict of interest.

## Publisher’s Note

All claims expressed in this article are solely those of the authors and do not necessarily represent those of their affiliated organizations, or those of the publisher, the editors and the reviewers. Any product that may be evaluated in this article, or claim that may be made by its manufacturer, is not guaranteed or endorsed by the publisher.

## Footnotes

1
https://www.synthego.com/blog/4-crispr-design-tools-to-improve-crispr-guide-rna-selection

2
https://www.arabidopsis.org/servlets/TairObject?type=stock&id=3001623338
